# The Prevalence and Determinants of Vitamin D Inadequacy among U.S. Older Adults: National Health and Nutrition Examination Survey 2007-2014

**DOI:** 10.7759/cureus.5300

**Published:** 2019-08-01

**Authors:** Carlos Orces, Carlos Lorenzo, Juan E Guarneros

**Affiliations:** 1 Rheumatology, Laredo Medical Center, Laredo, USA; 2 Rheumatology, University of Texas, San Antonio, USA; 3 Medicine, Universidad Anáhuac, Huixquilucan, MEX

**Keywords:** vitamin d, older adults, supplements

## Abstract

Background

Older adults (i.e., adults aged ≥ 60 years) are at higher risk of vitamin D deficiency compared to younger adults as a result of inadequate dietary vitamin D intake and limited exposure to sunlight. Thus, the present study aimed to describe the prevalence of vitamin deficiency and inadequacy among U.S. adults aged ≥ 60 years and the effect of vitamin D supplementation on 25, hydroxyvitamin D (25(OH)D) and its metabolites concentrations.

Methods

The present analysis was based on data from 6,261 participants in the National Health and Nutrition Examination Survey cycles 2007/2008 through 2013/2014. The prevalence of vitamin D deficiency and inadequacy was described according to demographic, behavioral, and health characteristics. Vitamin D deficiency was defined as 25(OH)D < 30 nmol/L; and vitamin D inadequacy was defined as < 50 nmol/L. Logistic regression models were assembled to examine the independent association of participants characteristics and the odds of having 25(OH)D inadequacy. Similarly, general linear models were used to assess the effect of vitamin D supplementation doses on 25(OH)D and its metabolites concentrations.

Results

The prevalence of 25(OH)D deficiency and inadequacy was 4.0% (standard error (SE), 0.4) and 17.4% (SE, 0.8), respectively. In general, the prevalence of 25(OH)D deficiency and inadequacy increased significantly among participants examined during the fall and winter months, women, non-Hispanic black patients, obese subjects, smokers, those physically inactive, and older adults with a daily vitamin D intake < 400 IU. After adjustment for potential confounders, subjects examined during the fall and winter months, females, non-Hispanic blacks, obesity, having a sedentary lifestyle, smokers, and a total vitamin D intake < 400 IU/day were variables significantly associated with increased odds of having vitamin D inadequacy. Notably, vitamin D supplement doses between 400 and 800 IU or > 800 IU/day were significantly correlated with higher 25(OH)D_3_ concentrations considered as sufficient.

Conclusion

25(OH)D inadequacy remains prevalent among U.S. older adults. Notably, optimal 25(OH)D_3_ concentrations were consistently seen among vitamin D supplement users. Despite this finding, nearly half of the participants did not take vitamin D supplements. Thus, vitamin D supplementation should be considered an effective strategy to maintain adequate 25(OH)D status among older adults.

## Introduction

Adults aged 60 years or older are at increased risk of vitamin D deficiency as a result of inadequate dietary vitamin D intake and limited sun exposure [1,2]. Aging is also associated with a decreased 7-dehydrocholesterol concentration in the epidermis and total production of pre-vitamin D3 after sunlight exposure [3]. The vitamin D endocrine system plays a primary role in calcium homeostasis, bone mineralization, and bone quality, and the vitamin D receptor is distributed throughout different tissues of the body. Observational studies among older adults have reported significant associations between lower serum hydroxyvitamin D (25(OH)D) levels and worse physical performance measures, disability, the metabolic syndrome, diabetes mellitus, and depression [4-6].

Although a recent analysis of the National Health and Nutrition Examination Survey (NHANES) showed no time trend in standardized 25(OH)D serum concentrations in the U.S. from 1988 to 2006, mean measured 25(OH)D concentrations were 5 to 6 nmol/L higher during 2007 to 2010. Further, the largest increases in 25(OH)D concentrations were seen among older adults, women, non-Hispanic white participants, and vitamin D supplement users [7]. A subsequent study of U.S. national levels of serum 25(OH)D during 2007 to 2010 described a crude prevalence of 25(OH)D deficiency and insufficiency of 5.7% and 22.0% among older adults, respectively [8]. Despite these facts, there are scarce data about the demographic and health determinants of vitamin D inadequacy among U.S. older adults since the 2011 Institute of Medicine (IOM) report on dietary reference intake values for vitamin D and the addition of vitamin D to the list of nutrients in the NHANES from 2007/2008 onwards [9,10]. In this context, the present study aimed to describe the nationwide prevalence and determinants of 25(OH)D inadequacy, and the effect of vitamin D supplements on 25(OH)D2 and 25(OH)D3 concentrations among older adults.

## Materials and methods

The NHANES is a biannual cross-sectional study conducted by the National Center for Health Statistics of the Centers for Disease Control and Prevention (CDC). The purpose of the NHANES is to collect data about the health, nutritional status, and health behaviors of the noninstitutionalized civilian resident population of the U.S. The NHANES data were obtained using a complex, multistage probability sampling design to select a sample representative of the U.S. civilian household population [11]. For this analysis, 7,859 participants aged 60 years and older were selected for the NHANES period 2007 to 2014. Those participants who had missing data on BMI (n = 514), dietary vitamin D intake (n = 945), and 25(OH)D concentrations (n = 990) were excluded, leaving a total sample size of 6,261 participants. Overall, participants with missing data were more likely to be women, had less than high school education, had a sedentary lifestyle, and reported to have fair to poor health. 

Characteristics of participants

The demographics file provides individual, family, and household-level information on the following topics: the six-month period when the examination was performed (November 1 through April 30 and May 1 through October 31), age, gender, race/ethnicity (Mexican American and other Hispanic were grouped as Hispanic, non-Hispanic white, non-Hispanic black, and other race), education (< high school, high school/GED equivalent, some college or Associate of Arts degree, college graduate or above). The ratio of family income to the poverty threshold as a measure of socioeconomic status was calculated, and families with a ratio < 1.00 were considered below the poverty level. In the mobile examination center, BMI was calculated as body weight (kg) divided by height (m^2^) and subjects were classified as underweight or normal weight (<25.0 kg/m^2^), overweight (25.0 to 29.9 kg/m^2^), or obese (≥30 kg/m^2^). Participants also reported their smoking status and were classified as current, former, and never smokers. Those who answered affirmatively to the question “In any one year, have you had at least 12 drinks of any type of alcoholic beverage?” were considered to consume alcohol. Self-reported general health condition was grouped as good to excellent or fair to poor. 

The Physical Activity Questionnaire was used to assess participants’ recreational physical activity status. Subjects were considered to perform recreational vigorous physical activity if they responded affirmatively to the question “Do you do any sports, fitness, or recreational activities that cause large increases in breathing or heart rate like running or basketball for at least 10 minutes continuously?” Likewise, subjects were considered to engage in moderate recreational activities if they affirmatively responded to the question “Do you do any moderate-intensity sports, fitness, or recreational activities that cause a small increase in breathing or heart rate such as brisk walking, bicycling, swimming, or golf for at least 10 minutes continuously?” The reported number of days and time in minutes spent performing vigorous or moderate recreational physical activity in the previous week were calculated. Based on the 2008 Physical Activity Guidelines for Americans, three levels of physical activity were created: 1) participants who engaged in ≥ 150 minutes/week of moderate activity, ≥ 75 minutes/week of vigorous activity, or ≥ 150 minutes/week of an equivalent combination were defined as physically active; 2) those who reported some physical activity, but not enough to meet the active definition (> 0 to < 150 minutes/week), were considered insufficiently active; and inactive if they reported no physical activity [3,12]. Participants were defined as having diabetes if they self-reported a physician's diagnosis of diabetes or had a glycosylated hemoglobin ≥ 6.5% [13]. Moreover, participants were asked, “Has a doctor or other health professional ever told you that you had arthritis, congestive heart failure, coronary heart disease, stroke, chronic bronchitis, or cancer?” Based on the subjects’ responses, a comorbidity score was created (0, 1, 2, ≥ 3).

Dietary vitamin D intake

The NHANES dietary intake data were used to estimate the types and amounts of foods and beverages consumed during the 24 hours prior to the interview, and to estimate intakes of nutrients from those foods and beverages. Vitamin D was added to the list of nutrients in 2007/2008, and the vitamin D values in this dataset reflect the sum of ergocalciferol and cholecalciferol content of foods reported by survey participants. The 24-hour dietary supplement interview was also collected following the 24-hour dietary recall. All NHANES examinees responding to the dietary recall interview were eligible for the dietary supplement and antacid use questions. We collected information on all vitamins, minerals, herbals, and other dietary supplements consumed during a 24-hour period, including the name and the amount of dietary supplement taken. These data were collected using similar methods during the same period; therefore, nutrients from all sources were easily combined to improve estimations of total nutrient intake and examine associations by nutrient source (e.g., from foods vs. supplements). For the present analysis, vitamin D intake from the dietary and supplementation component was combined to estimate the total daily dietary vitamin D intake. In the NHANES, vitamin D intake from food or supplements was reported in mcg/day and converted (1 mcg = 40 IU) into three IU/day categories (< 400 IU, 400 to 800 IU, and > 800 IU/day).

25(OH)D concentrations

The CDC standardized liquid chromatography-tandem mass spectrometry (LC-MS/MS) method was used for measurement of 25(OH)D for NHANES 2007 to 2010, which allows laboratories and surveys to compare 25(OH)D measurements. The CDC decided to develop an LC-MS/MS method traceable to the National Institute of Standards and Technology (NIST)-reference materials for NHANES, and used this method starting with NHANES 2007 to 2008 to “measure 25(OH)D3, 25(OH)D2, and the C3 epimer of 25(OH)D3. For the CDC LC-MS/MS method, total 25(OH)D (in SI units of nmol/L) was defined as the sum of 25(OH)D3 and 25(OH)D2 and excluded the C3 epimer of 25(OH)D3” [14]. However, according to the CDC, “due to rounding, the sum of 25(OH)D3 and 25(OH)D2 will not necessarily be equal to the total 25(OH)D.” The CDC recommends using the total 25(OH)D in SI units (nmol/L) measured directly by LC-MS/MS and converting this quantity to conventional units (1 nmol/L = 0.4066 ng/mL) if needed. This method has better analytical specificity and sensitivity compared to immunoassay methods, and fixed analytical goals for imprecision (≤10%) and bias (≤5%) [14].

Statistical analysis

The descriptive characteristics of the study population were reported as percentages and mean values with their respective standard errors (SE). The t-test and ANOVA for continuous variables were used to compare mean 25(OH)D concentrations across characteristics of participants. Similarly, the prevalence of 25(OH)D deficiency and inadequacy was reported according to the IOM 25(OH)D cutoff points relative to bone health (< 30 nmol/L and < 50 nmol/L, respectively) [9]. The chi-squared test was used to compare differences in the prevalence of 25(OH)D deficiency and inadequacy according to demographic, behavioral, and health characteristics. Subsequently, multiple logistic regression models were assembled to estimate the independent associations between six-month study period, sociodemographic characteristics (age, gender, race/ethnicity, BMI, education, and poverty level), lifestyle (smoking status, alcohol consumption, physical activity), health status (self-reported health, diabetes, and self-reported number of comorbidities), daily vitamin D intake (< 400 IU, 400 to 800 IU, and > 800 IU), and the odds of having 25(OH) inadequacy (< 50 nmol/L) while simultaneously adjusting for all variables in the study. Similarly, in a subgroup analysis, adjusted general linear models were created to examine the effect of vitamin D supplements use according to selected characteristics of participants on 25(OH)D and its metabolites concentrations. Statistical analyses were performed using SPSS Complex Sample software, Version 17.0 (SPSS Inc., Chicago, Illinois, USA) to incorporate constructed weights for the combined survey cycles and obtain unbiased, national estimates representative of the U.S. population.

## Results

A total of 6,261 participants with a mean age of 69.5 years (SE, 0.1) comprised the study sample, representing an estimated 56 million U.S. older adults during the 2007 to 2014 period. Overall, the mean daily vitamin D intake was 22.6 mcg (SE, 1.0), corresponding to 4.7 mcg (SE, 0.08) from food and 17.9 mcg (SE, 1.0) from supplements. Of note, 49.3% (SE, 0.9) of older adults were not taking vitamin D supplements. As shown in Table 1, lower mean 25(OH)D concentrations were seen among participants examined November 1 through April 30, men, Hispanics and non-Hispanic blacks, subjects with incomes below poverty level, smokers, those physically inactive, and older adults with fair to poor health. Likewise, participants with a total vitamin D intake or vitamin D supplements use < 400 IU/day had lower 25(OH)D concentrations than those who did not. 

**Table 1:** Mean 25(OH)D concentrations among U.S. older adults: the NHANES 2007-2014 *p < .005; **p < .0001 Abbreviations: AA, Associate of Arts degree; SE, standard error; NHANES, National Health and Nutrition Examination Survey; 25(OH)D, 25, hydroxyvitamin D.

	n	% (SE)	25(OH)D levels (SE)
Six-month time period			
Nov 1st to Apr 30th	2,753	38.4 (3.3)	73.6 (1.2)*
May 1st to Oct 31th	3,508	61.6 (3.3)	78.9 (0.8)
Age (years)			
60 - 69	3,138	53.6 (1.0)	75.1 (0.9)**
70 - 79	1,995	30.2 (0.7)	77.9 (1.0)
≥ 80	1,128	16.2 (0.7)	80.7 (1.5)
Gender			
Male	3,087	45.6 (0.6)	73.7 (0.7)**
Female	3,174	54.4 (0.6)	79.6 (1.0)
Race/ethnicity			
Hispanic	1,315	7.1 (0.9)	64.1 (1.1)**
Non-Hispanic white	3,344	80.6 (1.4)	79.9 (0.8)
Non-Hispanic black	1,231	7.9 (0.8)	60.3 (1.2)
Others	371	4.4 (0.4)	73.0 (2.2)
BMI (kg/m^2^)			
< 25	1,581	25.9 (0.8)	83.3 (1.3)**
25.0 to 29.9	2,282	36.3 (0.7)	78.1 (0.9)
≥ 30	2,398	37.8 (0.8)	71.3 (0.9)
Education			
Less than high school	1,961	20.4 (1.1)	70.0 (1.3)**
High school graduate	1,464	23.7 (0.8)	76.0 (1.2)
Some college or AA degree	1,562	28.8 (0.9)	78.6 (0.8)
College graduate or above	1,265	27.1 (1.3)	81.1 (1.1)
Income to poverty ratio < 1.00			
Yes	952	9.3 (0.6)	66.4 (1.2)**
No	4,779	90.7 (0.6)	78.0 (0.8)
Smoking status			
Never	3,038	49.1 (1.0)	78.1 (1.0)**
Former	2,430	40.3 (0.8)	77.6 (0.7)
Current	760	10.6 (0.5)	68.5 (1.9)
Alcohol use			
Yes	3,961	69.4 (1.1)	77.9 (0.8)
No	2,068	30.6 (1.1)	75.9 (1.1)
Physical activity status			
Inactive	3,857	56.5 (1.2)	72.7 (0.8)**
< 150 min/week	917	15.6 (0.6)	80.0 (1.3)
≥ 150 min/week	1,484	27.9 (1.0)	83.6 (1.3)
General health condition			
Good to excellent	4,356	78.4 (0.8)	79.2 (0.7)**
Fair to poor	1,902	21.6 (0.8)	68.5 (1.0)
Diabetes			
Yes	1,694	22.2 (0.8)	69.6 (0.9)**
No	4,337	77.2 (0.8)	78.9 (0.8)
Number of comorbidities			
0	2,048	31.4 (1.0)	74.9 (1.2)
1	2,462	40.1 (1.0)	77.5 (0.7)
2	1,176	21.0 (0.7)	78.9 (1.3)
≥ 3	449	7.4 (0.6)	78.2 (2.3)
Total daily vitamin D intake			
< 400 IU	3,287	46.4 (0.9)	62.8 (0.9)**
400 - 800 IU	1,300	22.0 (0.8)	80.0 (0.9)
> 800 IU	1,516	31.5 (1.0)	95.1 (0.8)
Vitamin D supplements/day			
< 400 IU	3,727	51.7 (0.9)	63.9 (0.9)**
400 - 800 IU	1,448	25.5 (0.8)	83.0 (0.9)
> 800 IU	1,086	22.8 (1.1)	99.5 (1.0)

The overall prevalence of 25(OH)D deficiency and inadequacy was 4.0% (SE, 0.4) and 17.4% (SE, 0.8), representing an estimated 2.2 and 9.7 million U.S. older adults during the study period, respectively. As shown in Table 2, the prevalence of 25(OH)D deficiency and inadequacy was increased among participants examined during the fall and winter months and in women, non-Hispanic blacks, obese subjects, smokers, those with a sedentary lifestyle, and subjects who reported taking a total vitamin D intake < 400 IU/day. 

**Table 2:** Prevalence of 25(OH)D deficiency and inadequacy among U.S. older adults: the NHANES 2007-2014 *p < .005; **p < .0001 Abbreviations: AA, Associate of Arts degree; SE, standard error; NHANES, National Health and Nutrition Examination Survey; 25(OH)D, 25, hydroxyvitamin D.

	25(OH)D < 30 nmol/L % (SE)	25(OH)D < 50 nmol/L % (SE)
Six-month time period		
Nov 1st to Apr 30th	5.4 (0.6)*	21.6 (1.2)**
May 1st to Oct 31th	3.1 (0.4)	14.8 (0.8)
Age (years)		
60 - 69	4.1 (0.5)	18.6 (1.0)
70 - 79	3.9 (0.5)	16.2 (1.2)
≥ 80	3.8 (0.6)	15.8 (1.3)
Gender		
Male	2.9 (0.3)**	15.3 (1.0)*
Female	4.9 (0.6)	19.2 (1.0)
Race/ethnicity		
Hispanic	5.2 (0.8)**	30.4 (2.1)**
Non-Hispanic white	2.7 (0.4)	13.5 (0.8)
Non-Hispanic black	15.6 (1.5)	42.9 (2.0)
Others	5.3 (1.5)	23.2 (2.8)
BMI (kg/m^2^)		
< 25	3.0 (0.5)**	13.9 (1.2)**
25.0 - 29.9	3.0 (0.4)	14.5 (1.0)
≥ 30	5.6 (0.6)	22.7 (1.1)
Education		
Less than high school	6.4 (0.8)**	25.1 (1.9)**
High school graduate	3.9 (0.5)	18.4 (1.4)
Some college or AA degree	3.6 (0.5)	15.8 (1.0)
College graduate or above	2.6 (0.5)	12.3 (1.1)
Income to poverty ratio (< 1.00)		
Yes	6.3 (0.9)**	29.4 (2.2)**
No	3.6 (0.4)	16.2 (0.8)
Smoking status		
Never	3.1 (0.3)**	16.3 (0.9)**
Former	3.8 (0.5)	15.2 (0.9)
Current	8.9 (1.4)	31.4 (3.0)
Alcohol use		
Yes	3.3 (0.4)*	15.8 (0.8)*
No	5.1 (0.7)	20.3 (1.4)
Physical activity status		
Inactive	5.4 (0.5)**	22.3 (1.0)**
< 150 min/week	3.5 (0.6)	15.1 (1.4)
≥ 150 min/week	1.5 (0.3)	8.8 (1.1)
General health condition		
Good to excellent	3.1 (0.4)**	15.0 (0.7)**
Fair to poor	7.2 (0.8)	26.1 (1.6)
Diabetes		
Yes	5.8 (0.7)**	25.0 (1.3)**
No	3.5 (0.4)	15.2 (0.8)
Number of comorbidities		
0	3.9 (0.5)	18.5 (1.5)
1	3.4 (0.4)	16.6 (0.8)
2	4.1 (0.7)	15.0 (1.3)
≥ 3	6.2 (1.3)	21.6 (2.5)
Total daily vitamin D intake		
0 - 400 IU	8.1 (0.8)**	33.3 (1.3)**
400 - 800 IU	0.9 (0.2)	6.9 (0.7)
> 800 IU	0.3 (0.1)	1.9 (0.4)

**Figure 1:**
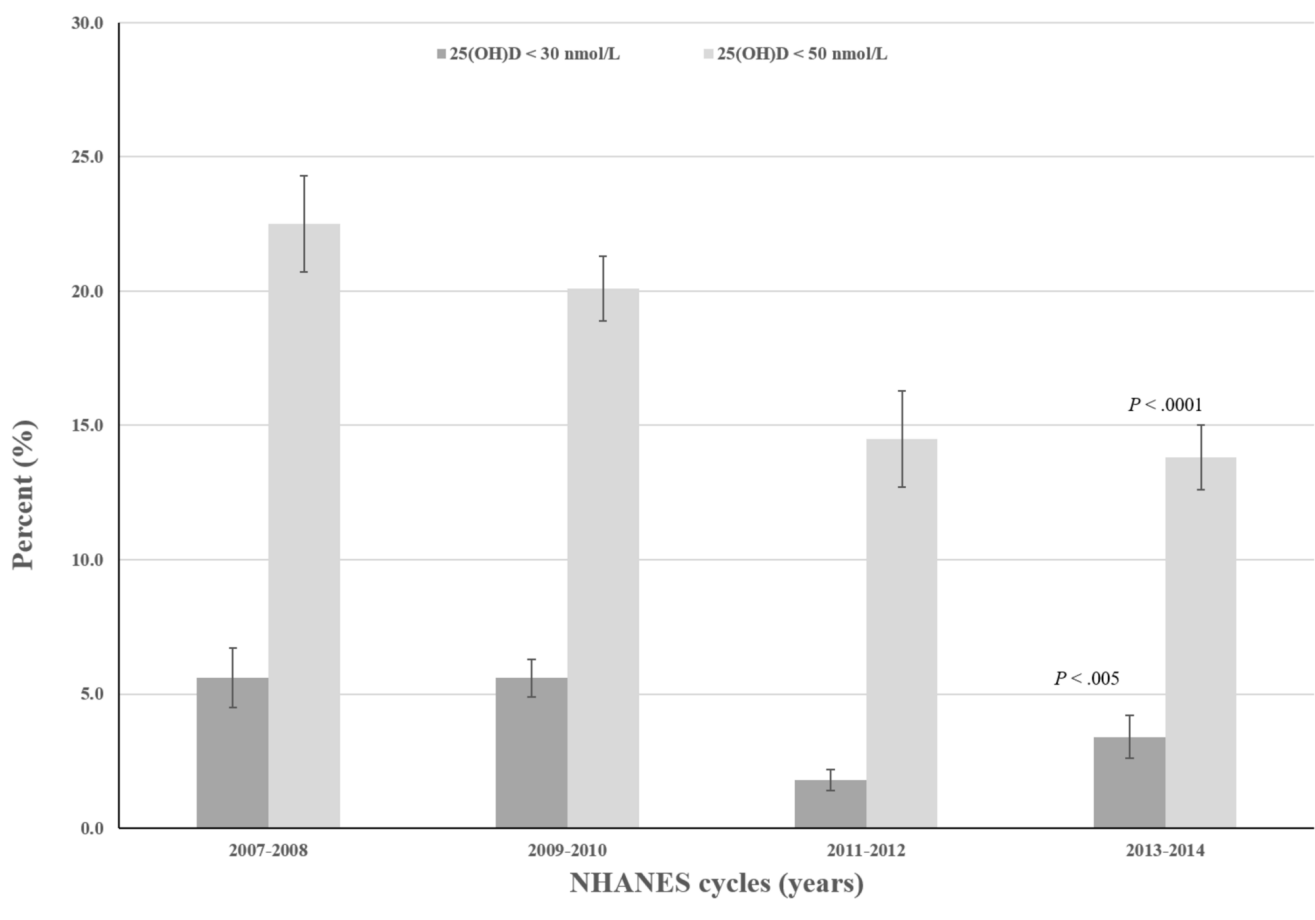
Trends in the prevalence of 25(OH)D deficiency and inadequacy among U.S. older adults

As shown in Figure 1, the prevalence of 25(OH)D deficiency and inadequacy significantly decreased among U.S. older adults from 5.6% and 22.5% in 2007/2008 to 3.4% and 13.8% in 2013/2014, respectively. 

As shown in Table 3, participants examined during the fall and winter months and obese subjects had about 50% higher prevalence of 25(OH)D inadequacy than those who did not. As expected, non-Hispanic blacks and Hispanics were 3.7-times and 1.4-times more likely to have 25(OH)D inadequacy compared with their non-Hispanic white counterparts, respectively. Moreover, participants with a sedentary lifestyle and status as a smoker had about two-fold higher odds of having 25(OH)D inadequacy compared with those physically active and of non-smoking status, respectively. Notably, older adults with a total vitamin D intake < 400 IU/day were 6.5 times more likely to have 25(OH)D inadequacy than those with intakes between 400 IU and 800 IU/day. 

**Table 3:** Characteristics of participants associated with increased risk of 25(OH)D inadequacy ^a^Models adjusted simultaneously for all variables shown in the table. ^b^Statistically significant odds ratios. Abbreviations: AA, Associate of Arts degree; OR, odds ratio; 25(OH)D, 25, hydroxyvitamin D.

	Crude OR (95% CI)	Adjusted OR (95% CI)^a^
Six-month time period		
Nov to Apr	1.58 (1.30, 1.91)	1.54 (1.21, 1.97)^b^
May to Oct	1.00 (ref)	1.00 (ref)
Age (years)		
60 - 69	1.00 (ref)	1.00 (ref)
70 - 79	0.84 (0.67, 1.05)	0.86 (0.67, 1.10)
≥ 80	0.81 (0.64, 1.04)	1.07 (0.82, 1.38)
Gender		
Male	1.00 (ref)	1.00 (ref)
Female	1.31 (1.08, 1.58)	1.57 (1.23, 2.02)^b^
Race/ethnicity		
Hispanic	2.80 (2.22, 3.52)	1.42 (1.02, 1.97)^b^
Non-Hispanic white	1.00 (ref)	1.00 (ref)
Non-Hispanic black	4.82 (3.83, 6.06)	3.77 (2.89, 4.90)^b^
Other race	1.93 (1.38, 2.71)	1.86 (1.20, 2.88)^b^
BMI (kg/m^2^)		
< 25	1.00 (ref)	1.00 (ref)
25.0 - 29.9	1.05 (0.85, 1.29)	0.95 (0.72, 1.24)
≥ 30	1.82 (1.43, 2.31)	1.59 (1.20, 2.12)^b^
Education		
Less than high school	2.38 (1.76, 3.21)	1.03 (0.73, 1.45)
High school graduate	1.60 (1.20, 2.14)	0.99 (0.72, 1.36)
Some college or AA degree	1.33 (1.06, 1.67)	0.86 (0.68, 1.10)
College graduate or above	1.00 (ref)	1.00 (ref)
Income to poverty ratio (< 1.00)		
Yes	2.14 (1.74, 2.64)	1.21 (0.97, 1.52)
No	1.00 (ref)	1.00 (ref)
Smoking status		
Never	1.00 (ref)	1.00 (ref)
Former	0.91 (0.76, 1.10)	1.08 (0.82, 1.40)
Current	2.35 (1.77, 3.10)	2.00 (1.39, 2.88)^b^
Alcohol use		
Yes	0.73 (0.61, 0.87)	0.87 (0.66, 1.14)
No	1.00 (ref)	1.00 (ref)
Physical activity status		
Inactive	2.99 (2.26, 3.97)	1.95 (1.38, 2.73)^b^
< 150 min	1.85 (1.33, 2.58)	1.34 (0.92, 1.94)
≥ 150 min	1.00 (ref)	1.00 (ref)
General health condition		
Good to excellent	1.00 (ref)	1.00 (ref)
Fair to poor	1.99 (1.68, 2.36)	1.14 (0.93, 1.40)
Diabetes		
Yes	1.86 (1.59, 2.17)	1.21 (0.96, 1.51)
No	1.00 (ref)	1.00 (ref)
Self-reported comorbidities		
0	1.00 (ref)	1.00 (ref)
1	0.87 (0.70, 1.09)	0.89 (0.70, 1.14)
2	0.77 (0.58, 1.02)	0.74 (0.51, 1.06)
≥ 3	1.21 (0.85, 1.71)	0.93 (0.62, 1.39)
Total daily vitamin D intake		
< 400 IU	6.74 (5.36, 8.47)	6.53 (5.03, 8.47)^b^
400 - 800 IU	1.00 (ref)	1.00 (ref)
> 800 IU	0.26 (0.15, 0.44)	0.23 (0.12, 0.43)^b^

As shown in Table 4, vitamin D supplementation use by selected participants’ characteristics was consistently correlated with increasing 25(OH)D3 concentrations. Indeed, after adjustment for possible confounders, subjects examined during the fall and winter months, obese participants, those physically inactive, and non-Hispanic blacks who were taking vitamin D supplements > 800 IU/day had on average 25(OH)D3 concentrations defined as sufficient (≥ 50 nmol/L). Notably, Hispanics and non-Hispanic blacks who reported taking vitamin D supplements > 800 IU/day had combined 25(OH)D2 and 25(OH)D3 concentrations of 91.6 nmol/L. 

**Table 4:** The effect of daily vitamin D supplements use on 25(OH)D metabolites concentrations among older U.S. adults Models adjusted for six-month time period, age, gender, race/ethnicity, education, socioeconomic status, BMI, smoking status, alcohol use, physical activity status, self-reported health, diabetes, number of comorbidities *p < .005; **p < .0001 Abbreviation: 25(OH)D, 25, hydroxyvitamin D.

	< 400 IU (ref)	400 - 800 IU	> 800 IU	< 400 IU (ref)	400 – 800 IU	> 800 IU
	25(OH)_2_levels (nmol/L)				25(OH)D_3_ levels (nmol/L)		
Six-month time period						
Nov 1st to Apr 30th	7.1 (1.2)	7.2 (0.8)	5.1 (0.8)	56.0 (1.1)	71.3 (1.8)**	91.3 (1.5)**
May 1st to Oct 31st	5.1 (0.6)	7.1 (0.6)*	7.4 (1.3)	61.7 (0.9)	77.1 (1.3)**	90.8 (1.6)**
Gender						
Male	3.9 (0.5)	6.5 (0.4)*	7.0 (1.1)*	60.9 (1.0)	73.7 (1.3)**	85.4 (2.3)**
Female	7.6 (1.2)	7.7 (0.7)	6.9 (1.1)	57.7 (1.2)	76.3 (1.4)**	93.4 (1.4)**
Race/ethnicity						
Hispanic	4.2 (0.5)	5.5 (0.8)	7.8 (2.1)	55.0 (1.3)	71.4 (1.5)**	83.8 (5.2)*
Non-Hispanic white	5.8 (1.0)	6.8 (0.4)	6.3 (0.9)	62.1 (1.1)	76.7 (1.1)**	91.9 (1.3)**
Non-Hispanic black	7.6 (0.9)	11.7 (2.2)	14.3 (3.2)*	42.1 (1.2)	61.1 (1.9)**	77.3 (4.1)**
Others	6.7 (1.9)	10.0 (2.6)	4.2 (1.6)	55.5 (2.1)	66.7 (4.1)*	100.9 (4.3)**
BMI (kg/m^2^)						
< 25	7.0 (1.4)	7.2 (0.8)	5.5 (0.9)	62.9 (1.7)	79.1 (1.8)**	98.0 (2.0)**
25.0 - 29.9	5.3 (0.7)	7.3 (0.6)*	6.8 (1.5)	62.4 (1.4)	76.9 (1.6)**	90.7 (1.8)**
≥ 30	5.8 (0.8)	6.9 (0.7)	7.4 (1.7)	54.0 (0.8)	70.5 (1.5)**	85.7 (2.5)**
Physical activity status						
Inactive	5.2 (0.7)	6.8 (0.6)	8.3 (1.5)	56.4 (1.0)	73.4 (1.3)**	88.2 (1.8)**
< 150 min/week	7.5 (2.0)	9.4 (1.5)	6.5 (2.6)	60.6 (1.4)	74.6 (2.1)**	92.7 (3.2)**
≥ 150 min/week	6.6 (1.0)	6.7 (0.5)	4.5 (1.0)	65.4 (1.7)	78.7 (1.8)**	94.6 (1.7)**

## Discussion

In this nationally representative sample, the prevalence of 25(OH)D deficiency and inadequacy was 4.0% and 17.4%, representing an estimated 2.2 and 9.7 million U.S. older adults during the study period, respectively. In general, participants examined during the fall and winter months, Hispanics, non-Hispanic blacks, smokers, those below poverty level, and particularly older adults with vitamin D intake < 400 IU/day had a higher prevalence of deficiency and inadequacy than those who did not. These findings are consistent with results from previous U.S. studies reporting similar determinants of vitamin D status [8,15]. Of relevance, the prevalence of 25(OH)D deficiency and inadequacy significantly decreased across the four continuous two-year survey cycles.

Similarly, Schleicher et al., using standardized 25(OH)D data to estimate temporal 25(OH)D status, also reported that 25(OH)D levels among U.S. adults aged 60 years and older increased from 58.4 nmol/L during 1988 to 1994 to 72.6 nmol/L in 2009 to 2010, a 24% increase [7]. A recent study demonstrated that individual vitamin D supplement use significantly increased among U.S. adults from 5% in 1999 to 2000 to 19% in 2001 to 2012. The authors in that particular study described that the highest increase in vitamin D supplement use was seen among subjects aged 65 years and older [16]. Since vitamin D supplement use is the main determinant of adequate vitamin D status among older adults, the increased use of vitamin D supplements, particularly among older adults over the past decade, may have accounted for the downward trend in the prevalence of vitamin D inadequacy found in the present study.

Although there is no universal consensus on the optimal 25(OH)D cutoff level that defines adequate 25(OH)D status, previous population-based studies among older adults, defining 25(OH)D inadequacy as a 25(OH)D cutoff level of < 50 nmol/L have reported prevalence rates ranging from 12.1% among subjects of diverse ancestry living in Toronto, Canada, 37.3% among participants in the Mexican Health and Aging Study, 21.6% among participants in an Ecuadorian survey, and 45% among participants of a Dutch study [17-20]. Moreover, the prevalence of 25(OH)D inadequacy found in the present study was similar to that reported among U.S. white participants aged 70 to 81 years [14]. 

Notably, 25(OH)D3 concentrations defined as sufficient (≥ 50 nmol/L) were seen among older Hispanics and non-Hispanic blacks who reported taking daily vitamin D supplements between 400 and 800 IU or higher doses. Likewise, participants examined during the fall and winter months, obese subjects, and those with a sedentary lifestyle maintained adequate 25(OH)D3 concentrations while taking vitamin D supplements. However, other sources of 25(OH)D3 such as food or endogenous production of the skin upon sunlight exposure may have contributed to higher 25(OH)D3 concentrations among vitamin D supplement users. Despite this evidence, an estimated 49% of older adults in the U.S. did not take vitamin D supplements, and only 25% reported taking daily vitamin D supplement doses of 400 to 800 IU. The 25(OH)D2 levels among non-Hispanic blacks markedly increased as vitamin D supplements also increase. In general, 25(OH)D2 is present in populations consuming sun-dried and ultraviolet B light-exposed mushrooms or 25(OH)D2 supplements. However, a recent analysis of the National Adult Survey in Ireland reported that 25(OH)D2 was present in the diet of the majority of adults at a variable but possibly nutritionally relevant levels [21]. Notably, vitamin D supplements use were significantly correlated with adequate 25(OH)D3 concentrations. Although we may postulate that this finding may be explained by a predominant use of cholecalciferol as the main source of vitamin D supplementation among U.S. older adults, the NHANES dietary file does not report the type of vitamin D supplements. However, the present study findings are consistent with results from previous studies conducted across different latitudes in which vitamin D supplementation was reported as a major determinant of vitamin D status among older adults [22,23]. 

U.S. older adults with incomes below the poverty level had a higher prevalence of 25(OH)D inadequacy compared with those who did not. A recent analysis of the NHANES 2007 to 2010 also reported that adults with high income were more likely to be vitamin D supplement users than those with medium or low incomes [24]. Likewise, low socioeconomic status has been previously associated with lower 25(OH)D concentrations [25]. Of the modifiable risk factors of vitamin D inadequacy, older adults with sedentary lifestyle had 1.9-fold higher odds of having 25(OH)D inadequacy compared with their physically active counterparts. In agreement with the present results, a recent analysis of the NHANES 2007 to 2012 demonstrated that older adults who are physically active had on average 8.1 and 7.1 nmol/L higher 25(OH)D and 25(OH)3 concentrations than those physically inactive, respectively [3]. The association of leisure-time physical activity and 25(OH)D concentrations has been explained by the effect of sunlight exposure on endogenous 25(OH)D3 synthesis during outdoor physical activity [3]. 

Shea et al. reported that U.S. white and black obese older adults had 56% and 37% higher odds of having 25(OH)D inadequacy compared with their normal-weight counterparts, which is consistent with the present study results [15]. The association between obesity and vitamin D deficiency has been explained by limited sun exposure, 25(OH)D sequestration within adipose tissue, and more recently lower 25(OH)D concentrations due to volumetric dilution [26]. Of note, smoking was a variable independently associated with inadequate vitamin D status. Previous reports have also shown that smoking is a negative predictor of 25(OH)D status among older adults. It appears that alterations in hepatic metabolism of vitamin D or 1-α-hydroxylation due to smoking could be a potential mechanism [27]. Alternatively, smoking may be a surrogate marker for adverse diet and lifestyle factors that diminish 25(OH)D, such as lower vitamin D intake [28]. In general, the number of self-reported comorbidities or diabetes was not associated with inadequate 25(OH)D levels. Although previous observational studies have demonstrated a consistent association of low serum 25(OH)D levels with prediabetes and diabetes, a causal relationship between 25(OH)D levels and the pathogenesis of type 2 diabetes mellitus has not been established [29].

The present study has several limitations. First, because the NHANES cross-sectional design, the temporal relationship of the study findings may not be established. Second, participants self-reported their demographics, lifestyle, and health characteristics, which may have been a source of recall bias. Third, sunlight exposure and sunscreen use were not reported among older adults in the NHANES. Fourth, the effect of latitude on 25(OH)D concentrations was unknown. However, serum 25(OH)D samples in the NHANES are collected from May through October in the northern U.S. and November through April in the southern U.S. [30]. Despite these limitations, the present study describes the prevalence and determinants of 25(OH)D inadequacy in a large nationally representative sample of older adults.

## Conclusions

Among older adults in the U.S., 25(OH)D inadequacy remains prevalent. Notably, optimal 25(OH)D3 concentrations were consistently seen among vitamin D supplement users. Despite this finding, about half of the study participants did not take vitamin D supplements. Thus, vitamin D supplementation should be considered as an effective strategy to maintain adequate 25(OH)D status among older adults. 
